# Supplementary material for: Imaging ferroelectric domains with a single-spin scanning quantum sensor
Source: Nat Phys. 2023 Feb 9;19(5):644–8. doi: 10.1038/s41567-022-01921-4 (PMC10185469; doi:10.1038/s41567-022-01921-4)
Supplement: Supplementary file 1 — Supplementary Sections 1–6 and Figs. 1–4. [file 41567_2022_1921_MOESM1_ESM.pdf]

nature physics

Letter

https://doi.org/10.1038/s41567-022-01921-4

Imaging ferroelectric domains with a single-spin scanning quantum sensor

In the format provided by the authors and unedited

CONTENTS

Supplementary Section 1: Theory of electric field gradiometry with NV centers 2
1.1. Small strain relative to electric field 2
1.2. Large strain relative to electric field 3
1.3. Off-axis magnetic field transition frequencies 4

Supplementary Section 2: Electric field gradient profiles 5
2.1. Monopole charge sheet domain wall 6
2.2. Dipole charge sheet domain wall 6

Supplementary Section 3: Sensitivity of NV electrometry 7

Supplementary Section 4: Improvements to spatial resolution and imaging speed 8

Supplementary Section 5: The NV reference frame 9

Supplementary Section 6: Additional figures 10

References 14

SUPPLEMENTARY SECTION 1: THEORY OF ELECTRIC FIELD GRADIOMETRY WITH NV CENTERS

In this section, the xyz coordinate system is taken as the NV center's coordinate system to simplify notation. The correction to the resonance frequencies (up to second order) for the ground state NV Hamiltonian subject to magnetic and electric fields is given by [1, 2]:

$$\omega_{\pm} = \omega_{\pm}^{(0)} + 2\pi k_{\parallel}\mathcal{E}_z \pm \left[\Omega(\vec{B},\vec{E},\vec{\xi}) - \Omega(\vec{B},0,\vec{\xi})\right], \tag{S1}$$

$$\Omega(\vec{B},\vec{E},\vec{\xi}) = \left[(\gamma_e B_z)^2 + (2\pi k_\perp \mathcal{E}_\perp)^2 - \frac{\gamma_e^2 B_\perp^2}{D} k_\perp \mathcal{E}_\perp \cos(2\varphi_B + \varphi_{\mathcal{E}}) + \frac{\gamma_e^4 B_\perp^4}{4(2\pi D)^2}\right]^{1/2}. \tag{S2}$$

$\omega_{\pm}^{(0)}$ is derived in Section 1.3, $\gamma_{\mathrm{e}} = 2\pi \times 28\,\mathrm{GHz/T}$ is the gyromagnetic ratio of the electron, $k_\parallel$ and $k_\perp$ are the on- and off-axis coupling for electric fields, $\vec{\mathcal{E}} = \vec{E} + \vec{\xi}$ is the combination of electric fields and strains ($\vec{\xi}$ is the strain written in units of volts per meter), $\tan(\varphi_B) = B_y/B_x$ and $\tan(\varphi_{\mathcal{E}}) = \mathcal{E}_y/\mathcal{E}_x$. Additionally, $\mathcal{E}_\perp = \sqrt{\mathcal{E}_x^2 + \mathcal{E}_y^2}$ and $B_\perp = \sqrt{B_x^2 + B_y^2}$. With $B_z = 0$ and $2\pi D \gg \gamma_{\mathrm{e}} B_\perp \gg 2\pi k_\perp \mathcal{E}_\perp$, $\Omega(\vec{B},\vec{E},\vec{\xi})$ can be simplified into:

$$\Omega(B_\perp,\vec{E},\vec{\xi}) = \frac{\gamma_e^2 B_\perp^2}{4\pi D} - 2\pi k_\perp \mathcal{E}_\perp \cos(2\varphi_B + \varphi_{\mathcal{E}}). \tag{S3}$$

For a general ac electric signal at frequency f, the vector components of $\mathcal{E}$ will look like $\vec{\mathcal{E}} = \vec{\mathcal{E}}_{\mathrm{dc}} + \vec{\mathcal{E}}_{\mathrm{ac}}\sin(2\pi f t)$. We assume that any dc component is solely attributed to internal strain ($\vec{\mathcal{E}}_{\mathrm{dc}} = \vec{\xi}$) as the dc electric field from the sample is entirely screened. The ac component, however, is solely attributed to the sample's signal ($\vec{\mathcal{E}}_{\mathrm{ac}} = x_{\mathrm{osc}}\partial_r \vec{E}$) since there is no ac strain. Moreover, the quantum phase acquired during an ac measurement is $\phi_\pm = \int_0^\tau g(t)\Delta\omega_\pm(t)dt$, where $\Delta\omega_\pm(t) = \omega_\pm(t) - \omega_\pm^{(0)}$ is the detuning. Next, we will show that in the low strain and high strain limits, it is possible to further simplify the expressions for the acquired phase.

1.1. Small strain relative to electric field

For small $\vec{\xi}$ compared to the electric field signal we can simplify $\mathcal{E}_\perp$ (electric field magnitude) and $\mathcal{E}_y/\mathcal{E}_x$ (related to the electric field angle) to get:

$$\frac{\omega_\pm(t) - \omega_\pm^{(0)}}{2\pi} = k_\parallel x_{\mathrm{osc}}\partial_r E_z \sin(2\pi f t) \mp k_\perp E_{\mathrm{ac}}|\sin(2\pi f t)|\cos\left(2\varphi_B + \varphi_{E_{\mathrm{ac}}}(t)\right) \tag{S4}$$

where E_{ac} takes on its definition from the main text and $\varphi_{E_{\mathrm{ac}}}(t)$ is a piecewise function,

$$\varphi_{E_{\mathrm{ac}}}(t) = \begin{cases} \arctan\left(\partial_r E_y / \partial_r E_x\right) & \text{if } \sin(2\pi f t) \leq 0 \\ \arctan\left(\partial_r E_y / \partial_r E_x\right) + \pi & \text{if } \sin(2\pi f t) > 0. \end{cases} \tag{S5}$$

While the square root term involving $\partial_r E_x$ and $\partial_r E_y$ produces a $|\sin(2\pi f t)|$ term, the absolute value is canceled by the changing electric field angle inside the cosine term. When the two-dimensional vector $[\partial_r E_x \sin(2\pi f t), \partial_r E_y \sin(2\pi f t)]$ passes through the origin (when $\sin(2\pi f t) = 0$), $\varphi_{E_{\mathrm{ac}}}(t)$ changes by π radians so the cosine term changes by a factor of -1. The on-axis coupling term $k_{\parallel}$ can be dropped as it is weak compared to $k_{\perp}$. As a result, the acquired phase for a general ac measurement sequence becomes

$$\phi_{\pm} = \mp \int_0^{\tau} g(t) 2\pi k_{\perp} E_{\mathrm{ac}} \cos\left(2\varphi_B + \varphi_{E_{\mathrm{ac}}}\right) \sin(2\pi f t) dt, \tag{S6}$$

where we have dropped the absolute term and let $\varphi_{E_{\mathrm{ac}}} = \arctan\left(\partial_r E_{y_{\mathrm{NV}}} / \partial_r E_{x_{\mathrm{NV}}}\right)$. Specifically for a gradiometry spin-echo sequence (equivalently CPMG-1 from ref. [3]), the phase is

$$\phi_{\pm} = \pm 4 k_{\perp} E_{\mathrm{ac}} \cos(2\varphi_B + \varphi_{E_{\mathrm{ac}}}) \sin^2(\pi f \tau / 2) / f, \tag{S7}$$

since $\int_0^{\tau} g(t) \sin(2\pi f t) dt = -2 \sin^2(\pi f \tau/2)/(\pi f)$ when the integral is centered on the zero crossing of the sine term. This equation is precisely what is shown in the main text. For multi-period gradiometry [3], the phase is

$$\phi_{\pm} = \mp 4 k_{\perp} E_{\mathrm{ac}} \cos(2\varphi_B + \varphi_{E_{\mathrm{ac}}}) \tau. \tag{S8}$$

1.2. Large strain relative to electric field

For large strains we get a different result from simplifying the electric field signal magnitude and angle expressions. From Eq. S1, we arrive at

$$\frac{\omega_{\pm}(t) - \omega_{\pm}^{(0)}}{2\pi} = k_{\parallel} \xi_z \mp k_{\perp} x_{\mathrm{osc}} \frac{\xi_x \partial_r E_x + \xi_y \partial_r E_y}{\xi_{\perp}} \sin(2\pi f t) \cos\left(2\varphi_B + \varphi_{\xi}\right). \tag{S9}$$

The numerator of the fraction on the right hand side in the equation above is equivalent to a two-dimensional dot product ($\vec{\xi}_{2D} \cdot \partial_r \vec{E}_{2D} = \xi_x \partial_r E_x + \xi_y \partial_r E_y = \frac{\xi_{\perp} E_{\mathrm{ac}}}{x_{\mathrm{osc}}} \cos(\varphi_{\xi} - \varphi_{E_{\mathrm{ac}}})$). With this expression, we can simplify Eq. S9 to

$$\frac{\omega_{\pm}(t) - \omega_{\pm}^{(0)}}{2\pi} = k_{\parallel} \xi_z \mp k_{\perp} E_{\mathrm{ac}} \cos(\varphi_{\xi} - \varphi_{E_{\mathrm{ac}}}) \cos(2\varphi_B + \varphi_{\xi}) \sin(2\pi f t). \tag{S10}$$

The acquired phase for a general ac measure sequence becomes

$$\phi_{\pm} = \mp \int_0^{\tau} 2\pi g(t) k_{\perp} E_{\mathrm{ac}} \cos(\varphi_{\xi} - \varphi_{E_{\mathrm{ac}}}) \cos(2\varphi_B + \varphi_{\xi}) \sin(2\pi f t) dt, \tag{S11}$$

and for the gradiometry spin-echo sequence, the phase is

$$\phi_{\pm} = \pm 4 k_{\perp} E_{\mathrm{ac}} \cos(\varphi_{\xi} - \varphi_{E_{\mathrm{ac}}}) \cos(2\varphi_B + \varphi_{\xi}) \sin^2(\pi f \tau/2)/f, \tag{S12}$$

In the high strain limit, it is possible to sense electric fields that are oriented along the direction of the perpendicular strain vector $[\xi_x, \xi_y]$, where $|\cos(\varphi_{\xi} - \varphi_{E_{\mathrm{ac}}})|$ is maximized. Effectively, the angular dependencies have shifted from $\cos(2\varphi_B + \varphi_{E_{\mathrm{ac}}})$ for small strains to $\cos(\varphi_{\xi} - \varphi_{E_{\mathrm{ac}}}) \cos(2\varphi_B + \varphi_{\xi})$ for large strains. Remarkably, the sensitivity remains the same, but there is a change in the direction in which the best sensitivity is achieved. An optimal scanning setup would orient the magnetic field angle to maximize $\cos(2\varphi_B + \varphi_{\xi})$.

1.3. Off-axis magnetic field transition frequencies

In the absence of electrical field and with a magnetic field applied in the NV center's xy-plane, the three-level ground state Hamiltonian [2] (with $\hbar = 1$) is,

$$H = \begin{bmatrix} 2\pi D & \frac{\gamma_e}{\sqrt{2}} B_{\perp} e^{-i\varphi_B} & 0 \\ \frac{\gamma_e}{\sqrt{2}} B_{\perp} e^{i\varphi_B} & 0 & \frac{\gamma_e}{\sqrt{2}} B_{\perp} e^{-i\varphi_B} \\ 0 & \frac{\gamma_e}{\sqrt{2}} B_{\perp} e^{i\varphi_B} & 2\pi D \end{bmatrix} \tag{S13}$$

where $B_x \pm i B_y = B_{\perp} e^{\pm i\varphi_B}$. The resulting energy eigenequation is $(\lambda - 2\pi D)(\lambda^2 - 2\pi D\lambda - \gamma_{\mathrm{e}}^2 B_{\perp}^2) = 0$, where λ are the eigenfrequencies. Solving this polynomial gives the energy levels corresponding to the eigenstates $|0\rangle$, $|-\rangle$, and $|+\rangle$. The eigenfrequencies are

$$\begin{aligned} \lambda_0 &= \frac{2\pi D - \sqrt{(2\pi D)^2 + (2\gamma_{\mathrm{e}} B_{\perp})^2}}{2} \\ \lambda_- &= 2\pi D \\ \lambda_+ &= \frac{2\pi D + \sqrt{(2\pi D)^2 + (2\gamma_{\mathrm{e}} B_{\perp})^2}}{2}, \end{aligned} \tag{S14}$$

and the resonance frequencies $\omega_{\pm}^{(0)}$ (given by $\omega_-^{(0)} = \lambda_- - \lambda_0$ and $\omega_+^{(0)} = \lambda_+ - \lambda_0$) are

$$\begin{aligned} \omega_-^{(0)} &= \frac{2\pi D + \sqrt{(2\pi D)^2 + (2\gamma_e B_{\perp})^2}}{2} \approx 2\pi D + \gamma_{\mathrm{e}}^2 B_{\perp}^2/(2\pi D) \\ \omega_+^{(0)} &= \sqrt{(2\pi D)^2 + (2\gamma_e B_{\perp})^2} \approx 2\pi D + \gamma_{\mathrm{e}}^2 B_{\perp}^2/(\pi D), \end{aligned} \tag{S15}$$

where an approximation was made for $\gamma_{\mathrm{e}} B_{\perp} \ll 2\pi D$.

SUPPLEMENTARY SECTION 2: ELECTRIC FIELD GRADIENT PROFILES

Here, we will formulate simplified expressions for the electric field (and electric field gradient) near ferrorelectric domains, starting from the electric field from a constant charged sheet. From Coulomb's law, the electric field from a finite charged sheet with surface charge density σ that extends from a_0 to a_1 in the x-direction and b_0 to b_1 in the y-direction is

$$E_x(x,y,z) = \frac{\sigma}{4\pi\epsilon_0}\left[\ln\left(\frac{\sqrt{(x-a_1)^2+(y-b_1)^2+z^2}-(y-b_1)}{\sqrt{(x-a_1)^2+(y-b_0)^2+z^2}-(y-b_0)}\right) - \ln\left(\frac{\sqrt{(x-a_0)^2+(y-b_1)^2+z^2}-(y-b_1)}{\sqrt{(x-a_0)^2+(y-b_0)^2+z^2}-(y-b_0)}\right)\right], \quad \text{(S16)}$$

$$E_y(x,y,z) = \frac{\sigma}{4\pi\epsilon_0}\left[\ln\left(\frac{\sqrt{(x-a_1)^2+(y-b_1)^2+z^2}-(x-a_1)}{\sqrt{(x-a_0)^2+(y-b_1)^2+z^2}-(x-a_0)}\right) - \ln\left(\frac{\sqrt{(x-a_1)^2+(y-b_0)^2+z^2}-(x-a_1)}{\sqrt{(x-a_0)^2+(y-b_0)^2+z^2}-(x-a_0)}\right)\right], \quad \text{(S17)}$$

$$E_z(x,y,z) = \frac{\sigma}{4\pi\epsilon_0}\left[\arctan\left(\frac{(x-a_1)(y-b_1)}{z\sqrt{(x-a_1)^2+(y-b_1)^2+z^2}}\right) - \arctan\left(\frac{(x-a_1)(y-b_0)}{z\sqrt{(x-a_1)^2+(y-b_0)^2+z^2}}\right) - \arctan\left(\frac{(x-a_0)(y-b_1)}{z\sqrt{(x-a_0)^2+(y-b_1)^2+z^2}}\right) + \arctan\left(\frac{(x-a_0)(y-b_0)}{z\sqrt{(x-a_0)^2+(y-b_0)^2+z^2}}\right)\right]. \quad \text{(S18)}$$

A domain wall can be constructed out of two charged sheets that meet at $x = 0$, propagate along the y direction and have opposite surface charge densities. For symmetry we will set $b_0 = -l_y$ and $b_1 = l_y$ (which will be useful in the limit of large l_y). Additionally, we can ignore the terms far away from $x = 0$, and focus on the xz-plane where $y = 0$. Here, the electric field at the domain wall is

$$E_x(x,0,z) = \frac{\sigma}{2\pi\epsilon_0} \ln\left(\frac{\sqrt{x^2+l_y^2+z^2}+l_y}{\sqrt{x^2+l_y^2+z^2}-l_y}\right)$$

$$E_y(x,0,z) = \frac{\sigma}{2\pi\epsilon_0} \ln\left(\frac{\sqrt{x^2+l_y^2+z^2}-x}{\sqrt{x^2+l_y^2+z^2}-x}\right) = 0 \quad \text{(S19)}$$

$$E_z(x,0,z) = \frac{-\sigma}{\pi\epsilon_0} \arctan\left(\frac{x l_y}{z\sqrt{x^2+l_y^2+z^2}}\right).$$

2.1. Monopole charge sheet domain wall

Simplifying the x and z components of Eq. S19 in the limit of large l_y, we arrive at

$$E_x^{\text{mono}} = \frac{\sigma}{2\pi\epsilon_0} \ln\left(1+\frac{4l_y^2}{x^2+z^2}\right)$$

$$E_z^{\text{mono}} = \frac{-\sigma}{\pi\epsilon_0} \arctan\left(\frac{x}{z}\right). \quad \text{(S20)}$$

While Eq. S20 is significantly simpler than Eq. S19, the logarithmic dependence on the length l_y is unwanted. However, if the spatial derivative is taken (along either the x- or z-direction), the dependence on l_y is removed (in the approximation for very large l_y). After taking the derivative in x and simplifying further, the electric field gradients are

$$\frac{\partial}{\partial x} E_x^{\text{mono}} = \frac{-\sigma}{\pi\epsilon_0} \frac{x}{x^2+z^2}$$

$$\frac{\partial}{\partial x} E_z^{\text{mono}} = \frac{-\sigma}{\pi\epsilon_0} \frac{z}{x^2+z^2}. \quad \text{(S21)}$$

2.2. Dipole charge sheet domain wall

Starting with Eq. S19, a surface of charged dipoles can be constructed by the addition of another monopole charge sheet domain wall with opposite charge at a distance d above the original. Succinctly, this can be represented as

$$\vec{E}^{\text{dipo}} = \vec{E}(x,0,z;\sigma) + \vec{E}(x,0,z-d;-\sigma). \quad \text{(S22)}$$

It is important to note that the original sheet is located at height of $z'=0$, the new sheet is located at $z'=d$ and the NV center (where the electric field is measured), is located at $z \gg d$. Thus, the distance between the NV and the bottom sheet is z and the distance

between the NV and the top sheet is $z - d$. With analogous derivations and simplifications as those shown in the previous section, the electric field components are

$$\begin{aligned} E_x^{\mathrm{dipo}} &= \frac{-\sigma d}{\pi \epsilon_0} \frac{z}{x^2 + z^2} \\ E_z^{\mathrm{dipo}} &= \frac{\sigma d}{\pi \epsilon_0} \frac{x}{x^2 + z^2}, \end{aligned} \tag{S23}$$

and the electric field gradients in the x-direction are

$$\begin{aligned} \frac{\partial}{\partial x} E_x^{\mathrm{dipo}} &= \frac{\sigma d}{\pi \epsilon_0} \frac{2xz}{(x^2 + z^2)^2} \\ \frac{\partial}{\partial x} E_z^{\mathrm{dipo}} &= \frac{\sigma d}{\pi \epsilon_0} \frac{z^2 - x^2}{(x^2 + z^2)^2}. \end{aligned} \tag{S24}$$

The difference in functional form between Eqs. S21 and S24 makes it possible to distinguish the type of surface charge distribution, despite the fact that one version includes an additional scaling parameter (d, the dipole length).

SUPPLEMENTARY SECTION 3: SENSITIVITY OF NV ELECTROMETRY

In a four-phase measurement [3, 4] the collected NV photoluminescence (PL) counts C_i are:

$$C_i = C_0 \left(1 - \frac{1}{2} \left[\epsilon + \epsilon_\tau \cos(\phi + \Phi_i) \right] \right), \tag{S25}$$

where C_0 is the number of counts for the $|0\rangle$ state, ϵ is the optical contrast (or Rabi contrast), ϵ_τ is the dephased optical contrast after time τ, ϕ is the acquired quantum phase over the evolution time τ and Φ_i is the final microwave $\pi/2$-pulse phase ($i = \{x, y, -x, -y\}$). From the four C_i measurements the acquired phase can be computed as

$$\phi = \arctan \left(\frac{\pm(C_{-y} - C_y)}{\pm(C_{-x} - C_x)} \right), \tag{S26}$$

where the $\pm$ in the numerator (denominator) is taken for even or odd number of π_y-pulses (π_x-pulses). Gaussian error propagation of Eq. S26 provides one method of estimating sensitivity.

First, we note that the best sensitvity is achieved by using multi-pulse measurements sequences and exploiting the increased sensor coherence time they provide [5]. Our best-effort sensitivity measurement use a dynamical decoupling XY8-3 sequence (24 decoupling π-pulses

in total) where phase was accumulated over 12 oscillation periods at $f \sim 196\,\mathrm{kHz}$ (equivalently $\tau \sim 61\,\mu\mathrm{s}$). With Eqs. S26 and S8 (setting the angular term to unity) the uncertainty in our NV electrometry measuement is

$$\sigma_{E_{\mathrm{ac}}} = \frac{1}{\sqrt{32}k_{\perp}\tau}\sqrt{\frac{C_x + C_{-x} + C_y + C_{-y}}{(C_x - C_{-x})^2 + (C_y - C_{-y})^2}}. \tag{S27}$$

Then, by normalizing that uncertainty by measurement time (following the $T^{-1/2}$ shot-noise measurement time scaling) we can estimate the sensitivity (Extended Data Fig. 3a,b).

We can also estimate the uncertainty by subtracting subsequent line scans (Extended Data Fig. 3c,d). In principle, taking the difference between neighboring pixels removes the electric field signal while amplifying the measurement noise by a factor of $\sqrt{2}$. Measurement time normalization is also needed here to produce a sensitivity estimate. The uncertainty estimates in Extended Data Fig. 3b,d are extracted from Gaussian fits and produce sensitivities ($0.24\,\mathrm{kV\,cm^{-1}\,Hz^{-1/2}}$ and $0.29\,\mathrm{kV\,cm^{-1}\,Hz^{-1/2}}$, respectively) that agree well with each other.

SUPPLEMENTARY SECTION 4: IMPROVEMENTS TO SPATIAL RESOLUTION AND IMAGING SPEED

Improvements in both the spatial resolution and imaging speed of NV electrometry are enabled by (i) increasing the measurement sensitivity, (ii) reducing the NV-sample distance, and (iii) increasing the signal at the NV center. By combining the improvements proposed below, it should be possible to perform NV electrometry with improved spatial resolution by a factor of two and a speed increase by an order of magnitude compared to our present work.

The sensitivity of the gradiometric detection scheme is improved by prolonging the evolution time τ (Eq. S27) while maintaining large optical contrast ϵ_τ (Eq. S25). This is achievable by using higher frequency oscillators and dynamical decoupling sequences with more π-pulses. With an oscillation frequency $f \sim 500$ kHz and a spin contrast which decays as $\epsilon_\tau = \epsilon \exp\left[-\left(\frac{\tau}{T_2 n^{2/3}}\right)^3\right]$, Ref. [5], where T_2 is the spin-echo coherence time, n is the number of π-pulses and $\tau = n/(2f)$ is the total evolution time, sensitivities of $\eta_{E_{\mathrm{ac}}} < 0.1\ \mathrm{kV\,cm^{-1}\,Hz^{-1/2}}$ are feasible with current scanning NV centers probes [3]. This alone can improve measurement speed by a factor of at least $5\times$ compared to our current sensitivity, as

the minimum detectable signal (which defines the noise level in SNR) decreases proportional to one over the square root of the pixel integration (or measurement) time ($T^{-1/2}$).

Spatial resolution is primarily determined by the NV-sample distance. That distance is composed of the NV implantation depth (from the tip surface to the NV) which should be approximately $5 - 10$ nm and the tip-sample distance (from the tip surface to the sample surface) that is determined by forces arising from our dynamic frictional (shear mode) AFM operation and feedback parameters. During gradiometry, we additionally retract the tip by ~ 20 nm to minimize tip-sample interaction that can affect the oscillation amplitude and phase of the tip [3]. This produces a total NV-sample distance of $z \sim 100$ nm. The retrack distance is non-optimal and can be eliminated through the use of AFM techniques that fix the oscillation amplitude and phase, such as frequency modulated (FM)-AFM [6]. Additionally, by tuning AFM feedback parameters, it is also possible to decrease the tip-sample distance by 10's of nanometers. Thus, gradiometry with NV-sample distances $z \leq 50$ nm should be possible, which would lead to an improvement in spatial resolution by 2×.

Both the use of higher oscillation frequencies and smaller NV-sample distances increase the signal at the NV center. While the increase in signal via a reduction in surface screening at higher frequencies is clearly demonstrated by our failure to image electric fields with dc protocols, the increase in electric field signal by decreasing the NV-sample distance is slightly more complicated to understand since the oscillation amplitude linearly affects the measured phase and is limited by the standoff distance ($x_{\text{osc}} \leq z$) to prevent spatial averaging [3]. The naive expectation is that the signal decreases with decreasing z. However, from Eqs. S21 and S24 it can be shown that the electric field signal maxima remains constant for monopole domains (for example $x_{\text{osc}}\partial_x E_z^{\text{mono}} \propto \frac{z^2}{x^2+z^2} \sim 1$ at $x = 0$ with decreasing z) and increases for dipole-like domains (for example $x_{\text{osc}}\partial_x E_z^{\text{dipo}} \propto \frac{z^3-x^2z}{(x^2+z^2)^2} \sim 1/z$ at $x = 0$ with decreasing z). For example, decreasing z from 100 to 50 nm would increase the electric field signal (and SNR as well) of our PZT sample by a factor of 2×.

SUPPLEMENTARY SECTION 5: THE NV REFERENCE FRAME

There are two common definitions for the NV reference frame. One reference frame defines $\hat{z}_{\text{NV}}$ as pointing from the nitrogen atom to the vacancy (N-to-V) and the second reference frame instead defines $\hat{z}_{\text{NV}}$ as pointing from the vacancy to the nitrogen (V-to-N). In both

cases, the $\hat{x}_{\mathrm{NV}}$ and $\hat{y}_{\mathrm{NV}}$ are defined the same way, where $\hat{x}_{\mathrm{NV}}$ points from the vacancy to one of the three nearest carbon atoms (while being perpendicular to $\hat{z}_{\mathrm{NV}}$) and $\hat{y}_{\mathrm{NV}} = \hat{z}_{\mathrm{NV}} \times \hat{x}_{\mathrm{NV}}$. In purely magnetic sensing applications, there is no practical difference between these definitions, since magnetic interactions can only determine the NV center's major symmetry axis (the z_{NV} axis), but not the $\hat{z}_{\mathrm{NV}}$ direction along that axis [7]. However, only the N-to-V definition should be adopted when translating NV electrometry measurements into the laboratory frame. This is because the electric field coupling constants ($k_{\parallel}$ and $k_{\perp}$), which are defined to be positive, were derived following the N-to-V convention [1, 2, 7].

The definition of $\hat{z}_{\mathrm{NV}}$ directly affects the definitions of $\hat{x}_{\mathrm{NV}}$ and $\hat{y}_{\mathrm{NV}}$, which has a major impact in the analysis of electric field data. For illustrative purposes, and without loss of generality, we choose to align $\hat{z}_{\mathrm{NV}}$ to point along the [111] crystal direction. Using the N-to-V definition the three nearest carbon atoms from the vacancy point along $[\bar{1}11]$, $[1\bar{1}1]$, and $[11\bar{1}]$ which would result in possible $\hat{x}_{\mathrm{NV}}$ definitions of $\frac{1}{\sqrt{6}}[-2, 1, 1]$, $\frac{1}{\sqrt{6}}[1, -2, 1]$, or $\frac{1}{\sqrt{6}}[1, 1, -2]$ and corresponding $\hat{y}_{\mathrm{NV}}$ definitions of $\frac{1}{\sqrt{2}}[0, -1, 1]$, $\frac{1}{\sqrt{2}}[1, 0, 1]$, or $\frac{1}{\sqrt{2}}[-1, 1, 0]$. Switching to the V-to-N definition (and keeping $\hat{z}_{\mathrm{NV}}$ along [111]) results in the three nearest carbon atoms from the vacancy to instead point along $[1\bar{1}\bar{1}]$, $[\bar{1}1\bar{1}]$, and $[\bar{1}\bar{1}1]$. This effectively inverts the resulting $\hat{x}_{\mathrm{NV}}$ and $\hat{y}_{\mathrm{NV}}$ definitions compared to the N-to-V definition. Consequently, the projection of the magnetic bias and electric fields onto the NV center's xy-plane differs by 180° (or π radians) and the acquired signal (proportional to $\cos(2\varphi_B + \varphi_E)$) thus differs by a minus sign. Therefore, laboratory frame electric fields with different polarities can be deduced by using different (and incorrect) NV reference frames. Careful attention is required for proper analysis and, for example, to correctly identify a given charge model.

Lastly, the specific choice of any of the three $\hat{x}_{\mathrm{NV}}$ possibilities (and corresponding $\hat{y}_{\mathrm{NV}}$) has no impact on the reconstruction of the laboratory frame electric field. Switching between different $\hat{x}_{\mathrm{NV}}$ choices is equivalent to a 120° (or $2\pi/3$ radians) rotation around the z_{NV} axis and the NV center's $C_{3\nu}$ symmetry results in the same NV behaviour. Specifically, the angular modulation term on the measured signal, $\cos(2\varphi_B + \varphi_E)$, is invariant when the magnetic bias field and electric field are shifted by 120° (or $2\pi/3$ radians).

SUPPLEMENTARY SECTION 6: ADDITIONAL FIGURES

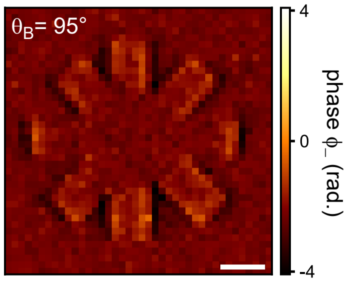

Figure S1. **NV electrometry on PZT with misaligned polar magnetic field angle.** Compared to the off-axis image (Fig. 2b of the main text) the signal amplitude is decreased and a large static offset is introduced by the 5° misaligned in the bias field. Scale bar, 1 μm.

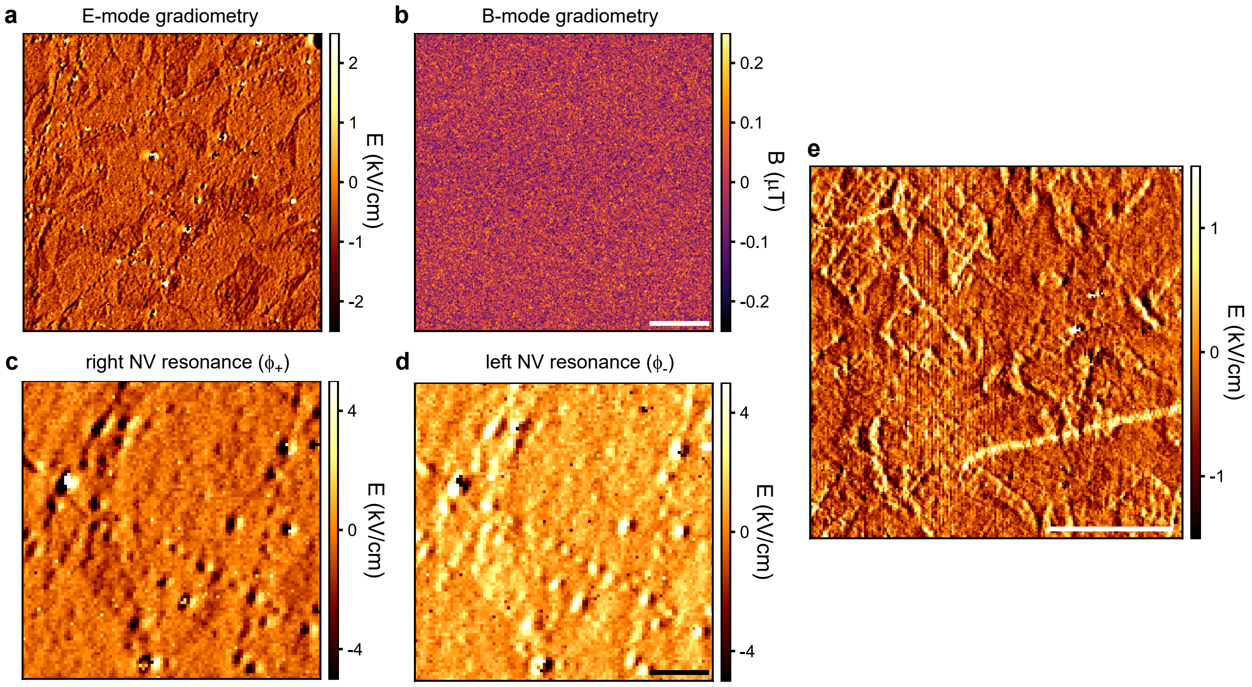

Figure S2. **Additional NV electrometry on $YMnO_3$.** **a**, Subsection of Fig. 4 of the main text. **b**, Gradiometry recorded with an on-axis bias field imaged over the same region in panel **a**. Here, the NV is sensitive to magnetic fields and none are detected. White scale bar, 5 μm. **c-d**, NV electrometry that simultaneously measured both transition frequencies ($\omega_{\pm}$), showing ferroelectric domains and charge accumulation with inverted contrast. Black scale bar, 1 μm. Panels **a-d** were recorded with tip #2. **e** NV electrometry recorded with tip #1 (used in Figs. 2 and 3 of the main text). This image reveals a similar domain structure to that of images recorded with tip #2, but different overall contrast compared to panel **a**.

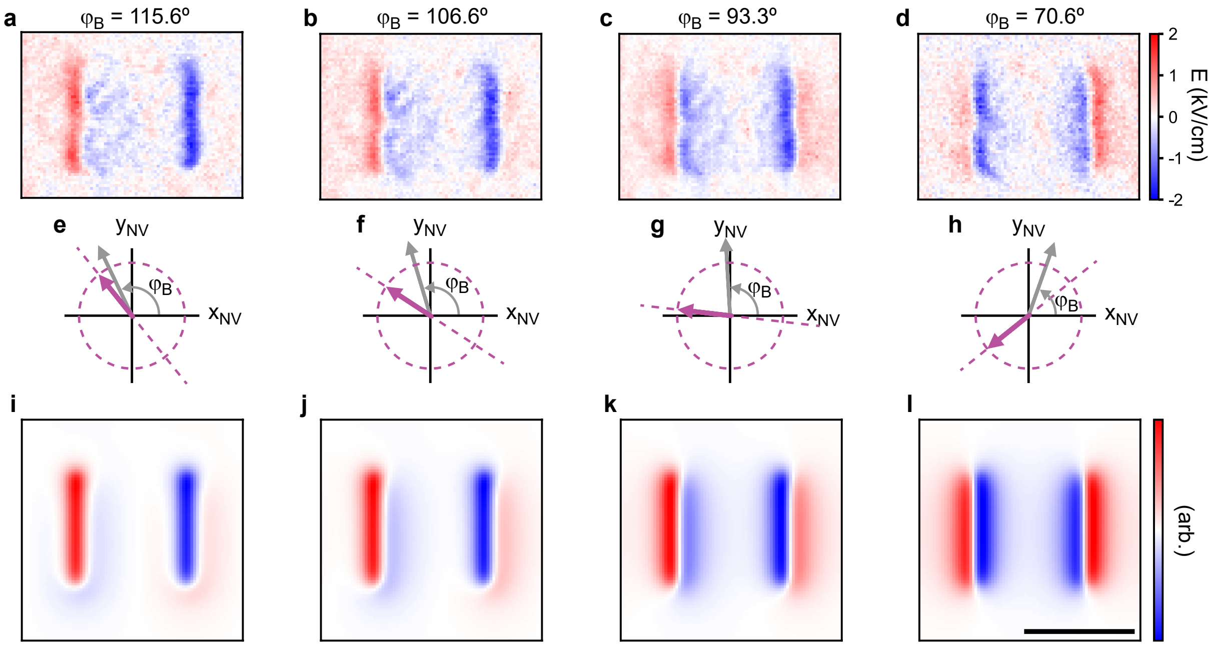

Figure S3. **NV electrometry with varying azimuthal (in-plane) magnetic field angle.** **a-d**, NV electrometry images taken over the pattern square domain shown in Fig. 2d of the main text with different in-plane magnetic field angles. The in-plane magnetic field angles are listed above. **e-h**, Schematics of the in-plane NV axes showing the magnetic bias field vector (gray) and detection axis (purple). **i-l**, Simulations of panels **a-d**. Scale bar, 1 μm.

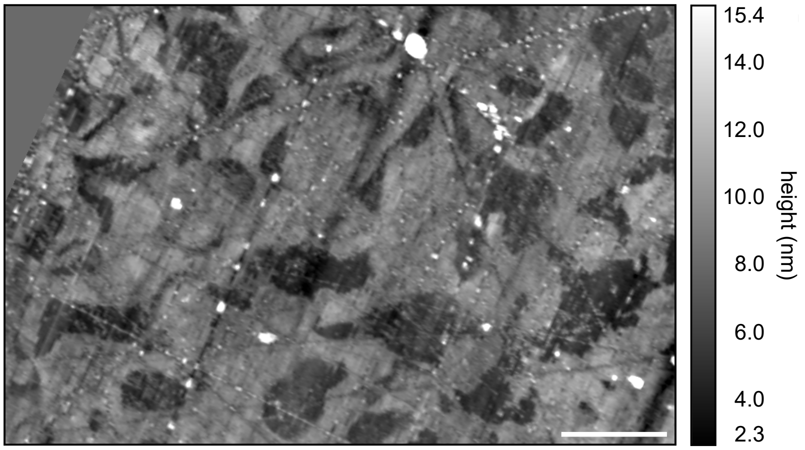

Figure S4. **Atomic force microscopy on $YMnO_3$.** AFM simultaneously acquired with the PFM image in Fig. 4b of the main text. Topographic contrast across domains is a result of the chemo-mechanical polishing, which is polarization dependent. Some colour-clipped regions (white) are examples of topographic crosstalk which produce artefacts in the PFM signal. Scale bar, 5 μm.

[1] F. Dolde, H. Fedder, M. W. Doherty, T. Noebauer, F. Rempp, G. Balasubramanian, T. Wolf, F. Reinhard, L. C. L. Hollenberg, F. Jelezko, and J. Wrachtrup, Electric-field sensing using single diamond spins, Nat. Phys. **7**, 459 (2011).

[2] M. W. Doherty, F. Dolde, H. Fedder, F. Jelezko, J. Wrachtrup, N. B. Manson, and L. C. L. Hollenberg, Theory of the ground-state spin of the NV^-center in diamond, Physical Review B **85**, 10.1103/physrevb.85.205203 (2012).

[3] W. S. Huxter, M. L. Palm, M. L. Davis, P. Welter, C.-H. Lambert, M. Trassin, and C. L. Degen, Scanning gradiometry with a single spin quantum magnetometer, Nature Communications **13**, 3761 (2022).

[4] M. L. Palm, W. S. Huxter, P. Welter, S. Ernst, P. J. Scheidegger, S. Diesch, K. Chang, P. Rickhaus, T. Taniguchi, K. Watanabe, K. Ensslin, and C. L. Degen, Imaging of submicroampere currents in bilayer graphene using a scanning diamond magnetometer, Physical Review Applied **17**, 10.1103/physrevapplied.17.054008 (2022).

[5] G. de Lange, Z. Wang, D. Riste, V. Dobrovitski, and R. Hanson, Universal dynamical decoupling of a single solid-state spin from a spin bath, Science **330**, 60 (2010).

[6] J. I. Kilpatrick, A. Gannepalli, J. P. Cleveland, and S. P. Jarvis, Frequency modulation atomic force microscopy in ambient environments utilizing robust feedback tuning, Review of Scientific Instruments **80**, 023701 (2009).

[7] M. W. Doherty, J. Michl, F. Dolde, I. Jakobi, P. Neumann, N. B. Manson, and J. Wrachtrup, Measuring the defect structure orientation of a single NV^- centre in diamond, New Journal of Physics **16**, 063067 (2014).
